# Combination of VSLAM and a Magnetic Fingerprint Map to Improve Accuracy of Indoor Positioning

**DOI:** 10.3390/s22239244

**Published:** 2022-11-28

**Authors:** Fang-Shii Ning, Mei-Hsin Chen, Shan-Gjie Lee, Yao-Chung Chen

**Affiliations:** 1 Department of Land Economics, National Chengchi University, Taipei 11605, Taiwan; 2 GIS Research Center, Feng Chia University, Taichung 40724, Taiwan

**Keywords:** indoor positioning technology, smartphone, magnetic fingerprint map, weighted k-nearest neighbor (WKNN), visual simultaneous localization and mapping (VSLAM), Oriented FAST and Rotated BRIEF (ORB), loose coupling, coordinate constraints

## Abstract

With the continual advancement of positioning technology, people′s use of mobile devices has increased substantially. The global navigation satellite system (GNSS) has improved outdoor positioning performance. However, it cannot effectively locate indoor users owing to signal masking effects. Common indoor positioning technologies include radio frequencies, image visions, and pedestrian dead reckoning. However, the advantages and disadvantages of each technology prevent a single indoor positioning technology from solving problems related to various environmental factors. In this study, a hybrid method was proposed to improve the accuracy of indoor positioning by combining visual simultaneous localization and mapping (VSLAM) with a magnetic fingerprint map. A smartphone was used as an experimental device, and a built-in camera and magnetic sensor were used to collect data on the characteristics of the indoor environment and to determine the effect of the magnetic field on the building structure. First, through the use of a preestablished indoor magnetic fingerprint map, the initial position was obtained using the weighted k-nearest neighbor matching method. Subsequently, combined with the VSLAM, the Oriented FAST and Rotated BRIEF (ORB) feature was used to calculate the indoor coordinates of a user. Finally, the optimal user’s position was determined by employing loose coupling and coordinate constraints from a magnetic fingerprint map. The findings indicated that the indoor positioning accuracy could reach 0.5 to 0.7 m and that different brands and models of mobile devices could achieve the same accuracy.

## 1. Introduction

Indoor positioning systems have diverse applications and substantial commercial value [1]. The use of indoor positioning systems is particularly beneficial in crowded areas, such as stores, stations, and large venues. Such systems are mainly used for personnel control and behavior analysis, and they improve management performance. In modern factory warehouses, indoor positioning systems are required for material and employee management, logistics tracking, and personnel supervision to ensure employee safety and smooth operations in factories. In long-term medical care facilities, indoor positioning systems can help accurately locate patients and older individuals that are in need in real time, thus facilitating the provision of immediate assistance and medical care.

The advantages of indoor positioning technologies are based on their cost, application, and accuracy. However, high precision is often accompanied with high costs or inefficiency, and a large area corresponds to lower precision. Thus, no single positioning technology can fully meet the aforementioned requirements. For example, GNSS signals cannot be received in an indoor environment because of signal masking. An inertial measurement unit (IMU) sensor generates positioning errors because of the accumulation of navigation information over time. The number of errors increases, and the accuracy decreases with the long-term use of an IMU sensor. Moreover, environmental factors limit the positioning accuracy of the monocular vision systems mounted on mobile devices; binocular vision is required between the point to be measured and the user. However, such vision cannot be occluded, and scale uncertainty exists. Thus, studies on indoor positioning technologies should focus on developing new positioning sources and effectively improving positioning accuracy and efficiency. Currently, a combination of technologies is employed in indoor positioning. Indoor positioning systems should meet certain requirements in accordance with the conditions of the application field or the habits of the users [2].

This study aimed to improve indoor positioning accuracy to within 1 m. We combined the magnetic fingerprint map and VSLAM methods. Although these individual methods have been done, the mixing of the two mentioned methods has not been done. We used the advantages of these two methods by coupling them. The coordinate constraint correction method combined the center position of the monocular VSLAM with the real-time trajectory coordinates obtained using the magnetic sensor to estimate the position. The findings were better than those of the individual methods. In this study, we used a combination of different sensors to reduce positioning errors. Because a magnetic field is recognizable and stable, it can be employed in indoor positioning. Therefore, a magnetic fingerprint map can be used in VSLAM. Mobile devices can be used to obtain optical images and indoor features, and VSLAM methods can be employed in indoor positioning. We used a smartphone as the experimental device. First, through the use of a preestablished indoor magnetic fingerprint map, the initial position of the user was determined by employing the weighted k-nearest neighbor (WKNN) matching method. Subsequently, we calculated the indoor coordinates of the user by employing a combination of VSLAM algorithms and the Oriented FAST and Rotated BRIEF (ORB) feature. Finally, the user’s optimal position was determined by using loose coupling and coordinate constraints from the magnetic fingerprint map. The findings indicated that the indoor positioning accuracy reached 0.5–0.7 m, and different brands and models of mobile devices exhibited the same accuracy.

The remainder of this paper was organized as follows: Section 2 introduces the related algorithms and experiments used in this study. Section 3 describes how VSLAM and a magnetic fingerprint map can be combined to improve the positioning accuracy of mobile devices. Section 4 presents an analysis and comparison of research findings. Section 5 presents a discussion of the results and concluding remarks.

## 2. Related Algorithms and Studies

### 2.1. Simultaneous Localization and Mapping

Algorithms are based on the data received from a sensor, and the position and orientation of the sensor are tracked while the environment map is constructed. Algorithms are considered to be VSLAM algorithms if a camera is used to obtain information on environmental factors. VSLAM algorithms can be used to determine the indoor positioning coordinates of a user by capturing images and calculating coordinate frames. Image processing is performed using a VSLAM algorithm. After image processing is conducted, feature point information can be obtained and used to estimate the user’s position and orientation. Image processing is divided into two components, namely feature point detection and feature point description, and it is performed to confirm the position and pattern of the feature points in an image.

**Figure 1:**
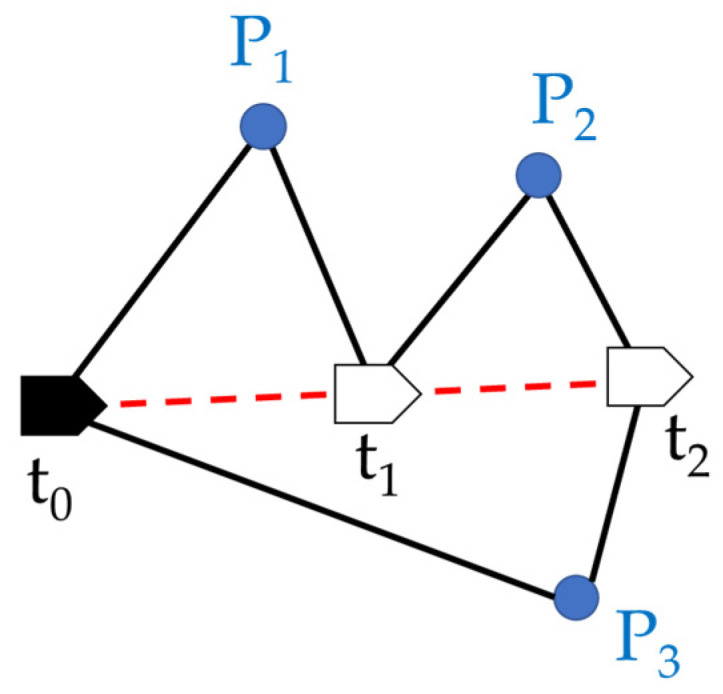
Constraint conditions for bundle adjustment.

Tareen et al. [3] and Karami et al. [4] tested various scenarios by using different image capture methods and comparing different feature point processing algorithms. Their findings indicated that ORB had the highest detection accuracy among various feature point detection techniques followed by speeded -up robust features (SURF). Scale-invariant feature transforms (SIFT) had the highest matching rate for numerous features. ORB maintained the highest running speed after error detection and matching. Rublee et al. [5] simultaneously extracted 1000 feature points from the same image. ORB maintained the rotation and scale invariance of the features, with a faster calculation time than SIFT and SURF. Thus, ORB is a better choice for real-time VSLAM than SURF or SIFT. In a VSLAM system, the mapping process is a front-end step that involves various stages from feature detection to motion estimation. Moreover, in the mapping step, the map is presented in the form of points and lines as presented in Figure 1. Bundle adjustment, which is the back-end step, is performed to determine the best continuous camera position and orientation in accordance with the known constraints, including the camera position [t_0_, t_1_, t_2_], spatial feature points [P_1_, P_2_, P_3_], and line segment constraints. Additionally, the areas between the points are composed of the camera observations of the feature points.

The currently available back-end processing methods are divided into two categories. Most of the back-end processing methods are based on filtering. The filtering-based methods mainly use the principle of Bayesian estimation to estimate the posterior probability of the user’s current position, orientation, and map feature position under the conditions that continual observation occurs and that the control information is known. Different filtering methods can be employed to estimate the posterior probability, including the Kalman filter (KF), extended KF, and particle filter. However, most of the filtering methods involve high computational resource costs. They require more time to obtain only part of the information that exists between adjacent photos, and they easily accumulate linear errors, thus causing difficulty in using the results to construct a map of large-scale scenarios [6,7]. Mur-Artal et al. [8] proposed the idea of using only the images near the current location to estimate a map based on the key frames. With this method, computing resources can be effectively used to optimize the bundle adjustment and improve the accuracy. In this method, bundle adjustment is performed to minimize reprojection errors with the help of the nonlinear least-squares technique. The bundle describes the line in which a point in three-dimensional space is projected onto the image plane. First, the point in three-dimensional space captured by a camera is projected onto the image plane. Subsequently, the image is used to triangulate the feature points, and the epipolar line geometry indicates the three-dimensional position. Finally, the calculated three-dimensional point coordinates and the relative motion of the camera are used to perform the second projection.

A reprojection error is the difference between the projection of the real three-dimensional space point on the image plane and the reprojection. In other words, a reprojection error is the difference between the pixels between the pixel point on the image and the virtual pixel point obtained from the calculated value. Because of factors such as accumulation errors and spatial changes, the calculated values differ from the actual values. In such cases, the sum of this difference should be minimized in order to determine the most favorable camera parameters and the three-dimensional space point coordinates. If the reprojection error formula is nonlinear, the initial solution for the relative camera movement is obtained by using visual odometry as the input of the least squares, and the optimal solution is obtained iteratively [9]. 

### 2.2. Magnetic Fingerprint Map

Similar to human fingerprints, magnetic fingerprints have unique characteristics. The magnetic field is not uniform in indoor environments, and fluctuations in magnetic field signals are caused by natural or artificial objects. The abnormal value of these magnetic fields can be used to determine a local magnetic field disturbance but not the overall stable magnetic field on a fingerprint map [10]. The magnetic intensities of different locations in indoor environments differ markedly. The effect of a building′s steel structure would not only cause errors in magnetic field positioning but would also distort the magnetic field fingerprint; this would result in unique indoor fingerprint characteristics and, thus, improve the positioning accuracy. 

The magnetic field data of an experimental area must be collected before a reference map of the magnetic field fingerprints is constructed. Traditionally, a single-point collection method has been employed in which a sensor is fixed at a certain position for a long time to collect data, and the average value is calculated as the magnetic intensity of the point. Then, different methods are used to construct a basic real-time map and to determine the magnetic value of the area for which data were not collected. The magnetic intensity of a given location can be more accurately determined using the single-point collection method; however, the construction of a database for a large range requires more time and higher human labor costs. Thus, this method is not suitable for updating data and is relatively inflexible [11].

Le Grand et al. [12] proposed a continuous acquisition method to solve the problem of low flexibility. In this method, the map of the magnetic field is built by covering the room twice in serpentines to form a square grid and recording the magnetic field along the lines parallel to two axes of the room. The built-in magnetic sensor continually collects magnetic field data pertaining to the relevant indoor environment. However, the moving speed differs each time the data are collected, providing results with different time series lengths. Therefore, the start and end points of collection trips should be fixed to ensure that the data collected have the same length as the raw data. Raw data are assigned a location on the basis of the length of their collection route.

Although the fingerprint determination process improves the efficiency of indoor magnetic field data collection, the lack of the measurement point density in some areas results in the absence of a spatial fingerprint. Therefore, according to the obtained discrete data and mathematical relationships, the magnetic field data of unknown points and regions are derived to solve the problem of discontinuous magnetic fingerprints [13]. When the indoor magnetic field anomaly is more prominent, the magnetic fingerprint will be more unique. In practical applications, the larger the number of fingerprint features is, the better the positioning is. Kriging interpolation is a spatial estimation method based on the principle of spatial statistics. It involves the use of the known observed values to estimate the random variables at any point in the random variable domain. In the same variable distribution, the observed values of N known points measured near the point to be measured are $Z\left(x_{1}\right)\;,Z\left(x_{2}\right)\;,...\;,Z\left(x_{N}\right)$. A linear combination of known observations is assumed to estimate the position of $x_{0}$ and the observational value of $\hat{Z}\left(x_{0}\right)$. $\lambda_{i}$ is the weight of the ith position′s observational value which is composed of the weighted sum of the data as shown in Equation (1).

(1) $$\hat{Z}\left(x_{0}\right)=\sum_{i=1}^{N\;}\;\lambda_{i}\cdot Z\left(x_{i}\right)\;$$

Compared with other interpolation methods, Kriging interpolation is more suitable for the construction of magnetic fingerprint maps because it is based on spatial correlation and uses the statistical relationship between measurement points to generate predictions. Kriging interpolation enables the generation of accurate predictions. After the complete consideration of the relationship between observed values, each observational value is assigned a weight, and the weighted average is used to obtain the estimated value. The subsequent research focused on three interpolation methods for the comparison of the preprocessing of magnetic fingerprint data. Correlation matching was first employed to determine the magnetic characteristics of the area that the user passed through, and a geomagnetic matching reference map was then constructed. When the user passed through these areas, the similarity in geomagnetism was used as the benchmark for matching, and the best matching point was obtained (i.e., the pedestrian′s position). The correlation matching method is simple. All numerical matches are independent of each other, and no calculation error is accumulated. Thus, there is no higher requirement for the initial point accuracy.

### 2.3. Hybrid Indoor Positioning

With the popularization of mobile devices, their internal sensors have gradually evolved, and their computing capabilities have considerably improved. Therefore, positioning technologies can be combined to improve indoor positioning accuracy. Qian et al. [14] combined the advantages of different sensors and applied them to structure from motion (SfM). They determined that complementarity between sensors can increase the stability of feature tracking, reduce the number of features required for camera motion, and robustly recover camera egomotion from image sequences belonging to different domains.

Corke et al. [15] divided the coupling method into loose and tight coupling. In loose coupling, different sensors are used as separate modules. Each module can obtain information on the position and orientation through calculations and can fuse them through filtering. The combination of different modules yields inconsistent update rates. In tight coupling, the intermediate data obtained from different sensors are processed using an optimization filter. In this process, the image features of the visual sensor need to be added to the feature vector, and the final system state vector has a higher dimension. The accuracy of tight coupling is higher than that of loose coupling. Table 1 presents a comparison of the two coupling methods.

Most studies used visual and inertial sensors as the two major modules in a coupling system, and these sensors can be combined in the fields of indoor positioning and robotics, including in virtual and augmented reality, positioning and navigation, position and motion estimation, and kinematic structure determination. Table 2 presents a comparison of the techniques used in previous studies. Different coupling frameworks have common problems. First, in the presence of different observational data, the state estimation of the sensor must be linearized in different reference coordinate systems. The four elements established through VSLAM are used as the model, and inertial sensing is used as the control unit. The real-time position and orientation are estimated using different filters. The second problem is related to the real-time correction of the relative position and orientation of a sensor. When coordinates are converted between different sensors, the consistency of the coordinates of the positioning system must be ensured. The third problem is related to the calibration and correction of the sensor parameters used to determine the scale and drift of relative measurements. In this study, to reduce the complexity of the system module and to maintain a high scalability, a loosely coupled framework was used as the basis for the combination of the positioning technologies.

In summary, all types of VSLAM systems can rapidly obtain three-dimensional coordinates in the user space and simultaneously construct an environmental point cloud map. However, because of the effect of accumulation errors, the user’s position deviates from the trajectory with an increase in time, leading to limitations in the positioning results. The user’s position when obtained from a magnetic fingerprint map exhibits a high sampling density based on data collection; thus, it provides high-precision spatial information and reflects the indoor space conditions in detail. However, considerable time and labor are required for this process. Moreover, the independent matching of the subsequent data points results in an abnormal trajectory of reentry and twists and turns. Because of the complex configuration of indoor space items and the movement of many pedestrians in an indoor space, signal-receiving equipment is often disturbed by environmental factors, causing difficulty in receiving signals from the users. Therefore, in the present study, mobile devices were used to obtain image information for the synchronization of map construction and visual positioning, and a magnetic sensor was employed to collect magnetic field data for the construction of an indoor magnetic fingerprint map. Finally, a loosely coupled algorithm was used to combine the two positioning results.

## 3. Methodology

### 3.1. Experimental Area

**Figure 2:**
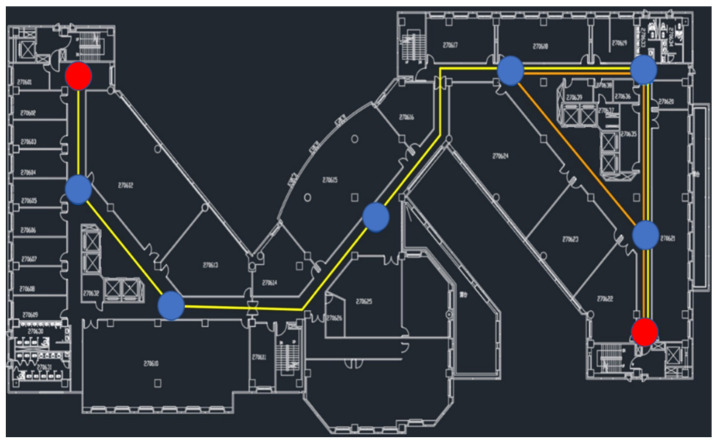
Building map and test route.

The sixth floor of the General Building of National Chengchi University was selected as the research area. Figure 2 presents the floor map and test route. This area was approximately 90 m in length and 40 m in width, and the movable corridor space was approximately 500 m^2^. The total length of the design route was 140 m, including 45° and 90° left and right rotations, respectively, and the corridor width was approximately 1.8 m. In Figure 2, red circles indicate the start and end points of the round-trip path, red and blue circles both show experimental check points used to detect coordinate errors when passing through the point, and the yellow line presents the experimental route.

A Xiaomi Mi 10 Lite mobile phone was used to collect positioning data. Table 3 lists its specifications. Many smartphone models have an Android operating system (OS), and the current global market share of this OS exceeds 80%. Moreover, the Android OS is more open than iPhone iOS; thus, we used the Android OS as the research tool in this study. Sensors used in the mobile device included an accelerometer, a magnetometer, and the phone′s in-built camera.

### 3.2. Study Process

The study process was mainly divided into training and positioning stages. The training stage involved establishing a magnetic field model and calibrating the camera of the mobile device. To build an indoor environmental magnetic fingerprint map database, the indoor environmental map was first obtained, and square grid paths were planned in accordance with ground tiles to determine the magnetic intensity. Furthermore, the interpolation method was used to estimate the values of points that were not present on the unplanned path. The camera calibration toolbox of MATLAB software (2020a) was used for processing, and the calibration result was used as the default value in the ORB-SLAM system.

The positioning stage involved the construction of a magnetic fingerprint map and the use of monocular VSLAM. The two built-in sensors in the mobile device were used to determine the dynamic position of the user. First, the magnetic sensor in the mobile device was used to obtain real-time magnetic field data on the planned path through fast acquisition. Subsequently, the IMU sensor was employed to obtain accelerometer data to determine the magnetic intensities of the horizontal and vertical directions. The single-point magnetic vector was used to solve the problem of nonunique features, and magnetic fingerprint matching was performed by comparing the findings of the nearest neighbor method with those of the prebuilt fingerprint model. Through the use of these methods, the user’s initial and subsequent positions were estimated.

A single-camera device was used to capture images while walking at the same speed in the same direction of the planned route. Image sequences were obtained by shooting the indoor environment. Sufficient indoor lighting was ensured. Feature point extraction and description methods were employed in accordance with the degree of similarity of feature points. After matching was completed, the motion relationship of sensors between adjacent photos was estimated. Continual information on position and orientation of single-camera device was collected using nonlinear optimization to improve the accuracy of positioning results and to obtain the best position estimates. Simultaneously, the closed-loop method was used to determine whether single-camera device had passed the previous position as well as to determine the difference between the two results and to restrict the position; these processes could solve the problems of accumulation errors and positioning drift.

**Figure 3:**
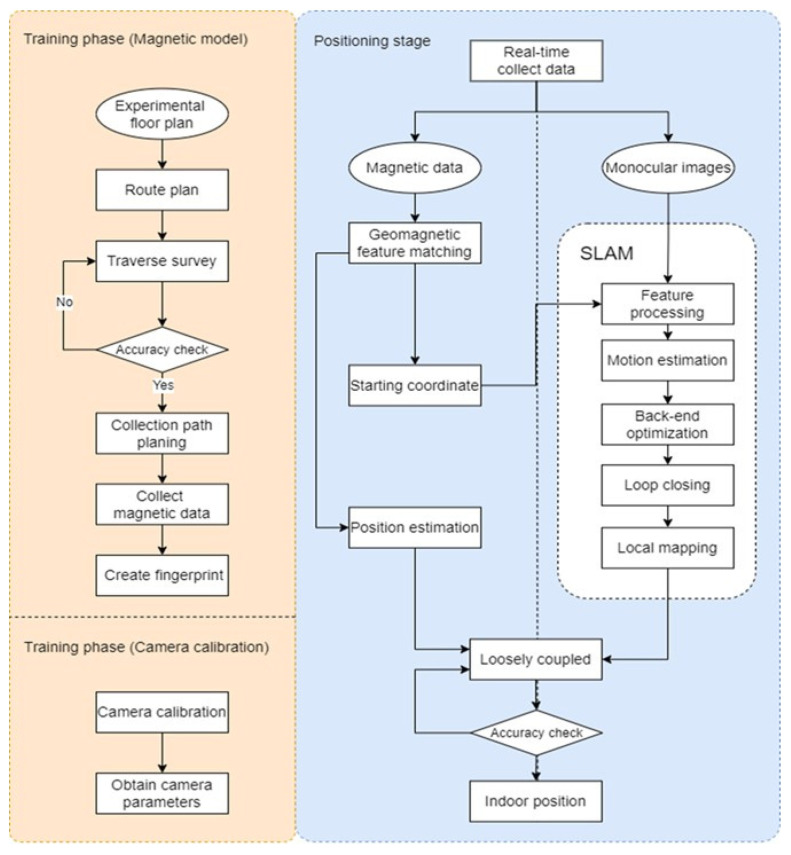
Research process (**left** represents training stage; **right** represents positioning stage).

Finally, the coordinate constraint correction method was used to combine the center position of the monocular VSLAM with real-time trajectory coordinates obtained using the magnetic sensor to optimize the user′s position. Figure 3 presents the flowchart of the training stage (left) and positioning stage (right).

### 3.3. Relative Matching Method

In this study, continuous collection and interpolation methods were used to generate a complete indoor magnetic fingerprint map, and the data were tested using the nearest neighbor (NN), k-nearest neighbor (KNN), and WKNN [20] algorithm. The NN algorithm is a deterministic algorithm mainly used for classification and fingerprint matching. It calculates the similarity between magnetic intensities determined in real time and those obtained from the preestablished magnetic fingerprint map. The point position with the highest similarity fingerprint was used as the final positioning coordinate. The user could obtain the magnetic vector $\left(M_{V}^{i},M_{H}^{i}\right)$ from the vertical direction and the horizontal direction in the experimental area. The distribution set of each point in the fingerprint database was $\left\{(M_{V}^{1},M_{H}^{1}),(M_{V}^{2},M_{H}^{2})\ldots (M_{V}^{j},M_{H}^{j})\right\}$. The Euclidean distance $L_{\mathrm{ij}}$ between vectors was calculated using Equation (2), and the minimum distance corresponding to the coordinate vector was used as the output. The NN algorithm is easy to implement. Its low computational cost is suitable for environments with large differences in fingerprint data.

(2) $$L_{\mathrm{ij}}=\sqrt{{\left(M_{V}^{i}-M_{V}^{j}\right)}^{2}+{\left(M_{H}^{i}-M_{H}^{j}\right)}^{2}\;}\;\;$$

The KNN is based on the NN algorithm and is used to identify K reference points closest to the magnetic vector measured in the positioning stage in the fingerprint database as well as to calculate the average value of coordinates corresponding to these vectors. Equation (3) presents the calculation formula. Different from the reference point selected using the NN algorithm, that selected using the KNN algorithm indicates the approximate area of the representative positioning point. The positioning accuracy was improved through averaging. $\left(X_{i},Y_{i}\right)$ represents the corresponding position of the ith magnetic field data in all vectors, and $\left(\bar{X},\bar{Y}\right)$ is the final positioning result obtained using the KNN algorithm.

(3) $$\mathrm{KNN}\left(\bar{X},\bar{Y}\right)=\frac{1}{K}\sum_{i=1}^{K}\left(X_{i},Y_{i}\right)\;$$

The WKNN algorithm differs from the KNN algorithm. It directly averages K positions to multiply each position coordinate vector by a weighting coefficient. As presented in Equation (4), the Euclidean distance between the fingerprints of the positioning points was used as the weighting basis. $L_{\mathrm{ij}}$ is the Euclidean distance of the magnetic intensity between the measuring point j and the point i in the magnetic fingerprint database.

(4) $$\mathrm{WKNN}\left(\bar{X},\bar{Y}\right)=\frac{\sum_{i=1}^{K}\frac{1}{L_{\mathrm{ij}}}\left(X_{i},Y_{i}\right)}{\sum_{i=1}^{K}\frac{1}{L_{\mathrm{ij}}}}\;\;$$

### 3.4. ORB-SLAM

**Figure 4:**
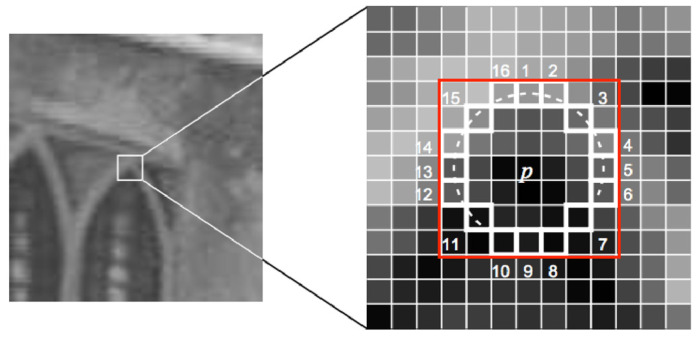
Schematic of FAST key point detection.

The ORB feature is used for image feature matching in VSLAM. Its main advantage is that it can maintain the scale and rotation inconvenience of feature description. The ORB feature is more computationally efficient compared to other feature algorithms, including key point and feature description. Oriented FAST key point extraction is an improved method based on FAST corner point feature extraction. The original FAST key point extraction is performed at places where the gray value of the local image changed significantly. This method involves the use of a pixel to compare it with adjacent pixels; a corner point is determined when the difference in the gray value exceeds the threshold value. Figure 4 presents a schematic of FAST key point detection [21]. A pixel p is randomly selected in the image with the gray value $I_{p}$, and a threshold value T is set. The pixel p is considered to be the center, and a total of 16 pixels on a circle with a radius of 3 pixels are selected. When three of the pixel gray values with serial numbers 1, 5, 9, and 13 on the circle are greater than $I_{p}+T$ or less than $I_{p}-T$ at the same time, the pixel is determined to be a feature corner. 

When the FAST feature points detect corner points, it only distinguishes the difference in the gray value between pixels but does not indicate the directionality or uneven distribution of pixels. In addition, when the same scene is photographed at different distances, the results of object recognition are affected. When viewed as feature points, the reduction in distance may produce different discriminant states. To solve these problems, ORB adds a description of scale and direction. Scale invariance is created by constructing an image pyramid. Corner detection is performed on images of different resolutions by using the intensity centroid method to determine the rotation angle of feature points. According to the image concept, the bottom layer is the original image, and each upper layer and the image are scaled at a fixed ratio. Thus, images of multiple resolutions are obtained, and the smaller scale can be regarded as a scene shot from a distance. In the feature matching algorithm, images with different resolution layers can be matched to achieve scale invariance.

The direction description is based on the calculation of the centroid of the gray value of the image near the feature point. That is, the gray value is used as the weight center to determine the degree of rotation. First, with the selection of an image block B, its moment can be expressed as Equation (5). The centroid C of the image block can be determined using the aforementioned moment. The geometric center O and the centroid of the image block are connected to obtain a direction vector $\bar{\mathrm{OC}}$. The direction of the feature point can be defined using Equation (6).

(5) $$m_{\mathrm{pq}}=\sum_{x,y\in B}x^{p}\cdot y^{q}\cdot I\left(x,y\right),\;p,q=\left\{0,1\right\}$$

(6) $$C=\left(\frac{m_{10}}{m_{00}},\frac{m_{01}}{m_{00}}\right),\theta =\tan^{-1}\left(\frac{m_{01}}{m_{10}}\right)$$

Because BRIEF is only a feature description, the position of the feature point needs to be determined first. Therefore, after the position of the feature point is found by using the aforementioned ORB feature point detection algorithm, the feature description of each point is calculated. In the range of feature points, BRIEF is used to establish feature descriptions. On the basis of the storage method of the binary system, after a certain number of sampling comparisons, a series of encoded messages are obtained, that is, image messages described by BRIEF. First, Gaussian filtering is performed on the image to reduce image noise interference and to use the feature point as the center. With the use of an S × S window, N pairs of random points are selected in the window, and N is usually set to 256. The gray values of the pixels are compared, as shown in Equation (7).

(7) $$\{\begin{array}{c} p>q:\;1\\ p<q:\;0 \end{array}$$

where 0 and 1 are defined by combining two random pixels near the key point into multiple point pairs $\left(p_{1},q_{1}\right)\left(p_{2},q_{2}\right)...\left(p_{n},q_{n}\right)$. The values p and q represent the gray values of the random point pair. If p is greater than q, the value is 1; otherwise, the value is 0. After a series of extractions, a series of binary codes [1,0,…,1] can be obtained which indicate the description of feature points. Any change in light and dark places does not affect the results if the relationship is maintained, so the lighting problem can be solved. Subsequently, a set of 256-bit binary codes is obtained for feature points in the image. Subsequently, two images with similar or overlapping parts are matched. When the number of identical elements for the corresponding bits of the two coding sequences is less than 128, the feature points are not the same. If the number of corresponding bits of the feature code on the image is the largest, the matching of the feature point is regarded as complete.

### 3.5. Coupling and Constraining System

The weighted fusion process uses the root mean square error (RMSE) as the weight basis, as presented in Equation (8). The RMSE is the squared sum of the deviation of the observed value and the true value of the average root; it can be used to determine the average difference between the observed and true value. Because the RMSE is sensitive to large or small errors in a set of measurements, it can effectively reflect the positioning accuracy.

(8) $$\mathrm{RMSE}=\sqrt{\frac{\sum_{i=1}^{n}{\left(X_{\mathrm{obs},i}-X_{\mathrm{tru},i}\right)}^{2}}{n}}\;,{\;\mathrm{where}\;X}_{\mathrm{obs},i}-X_{\mathrm{tru},i}=\sqrt{\Delta x_{i}{}^{2}+\Delta y_{i}{}^{2}}$$

where RMSE is the root mean square error of distance between observational coordinate and true coordinate; n is the number of measurements; $X_{\mathrm{obs},i}$ is the ith observational coordinate; $X_{\mathrm{true},i}$ is the ith true coordinate; $\Delta x_{i}$ is the ith error of x-axis coordinate; $\Delta y_{i}$ is the ith error of y-axis coordinate.

In addition to combining two positioning data in accordance with the time series, Ning et al. [22] proposed the use of scene constraints for indoor navigation and positioning, the determination of the time point of correction by evaluating the azimuth angle and turning characteristics, and the correction of coordinates to a known point to estimate the initial value of a pedestrian′s position. This study extended this proposition and used the ORB-SLAM method for calculating coordinates. When passing through the turning point, the user′s coordinate, obtained from magnetic fingerprint map, was regarded as the new starting position and was used as the basis for the ORB-SLAM conversion. The 4-point congruent set (4PCS) cloud matching algorithm regarded the position of the key frame in the image coordinate system and the starting point obtained using magnetic field positioning as point clouds for matching. The conversion parameters between the two were obtained to solve the problems of the ORB-SLAM scale and turning. To ensure that magnetic field positioning restricted visual positioning, different positioning systems were combined using the two aforementioned coupling methods.

## 4. Results and Discussion

The results were divided into two sections. The first section describes the results of the camera calibration of the mobile device and the establishment of the magnetic field model in the training phase. The second section describes the results of the magnetic field positioning method and the ORB-SLAM method used for coordinate constraint corrections. In addition, the accuracy of the positioning results is discussed. 

### 4.1. Analysis of the Results of the Training Phase

The calibration data in this study were obtained from images captured by the built-in camera of the Xiaomi Mi 10 Lite smartphone. During the shooting process, checkerboard calibration was used as the center. In every set, a total of 18 images were shot horizontally and vertically in nine directions around and directly above the center. Three sets of data were used for testing. The camera calibration toolbox for the MATLAB 9.8 software (R2020a) was used for processing, and the calibration result was used as the default value in the ORB-SLAM system. During the image collection process, the anti-shake feature of the mobile phone was turned off to prevent the automatic correction of the image. In addition, we ensured that the calibration mark remained flat and that the surrounding area remained white. We improved the image contrast and calibration effect and increased the accuracy of corner extraction by using the checkerboard calibration mark with different lengths and widths. Table 4 presents the calibration results, where f is the focal length; $x_{p}{\;\mathrm{and}\;y}_{p}$ are the principal points of the image; $K_{1},K_{2},{\mathrm{and}\;K}_{3}$ are the radiation distortion parameters; and $P_{1}{\;\mathrm{and}\;P}_{2}$ are the radiation distortion parameters.

**Figure 5:**
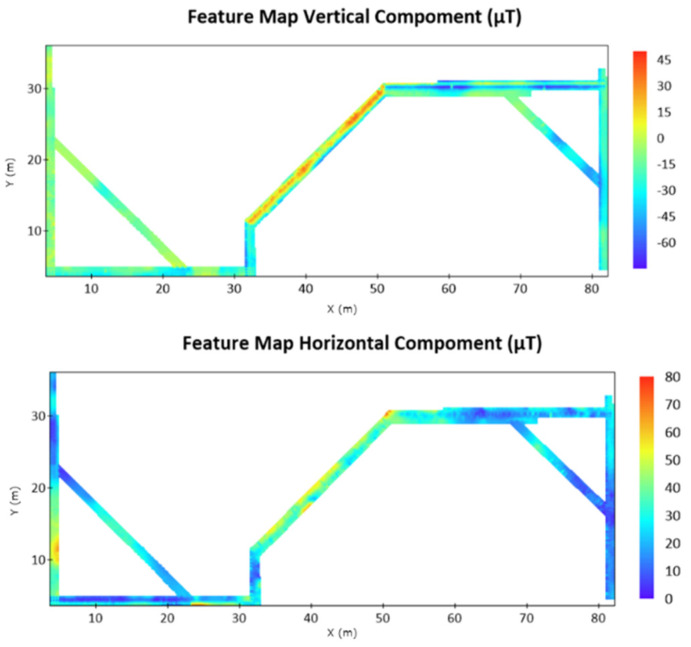
Results related to the collected magnetic field (after interpolation).

Data sampling was performed on the basis of the planned route. The Xiaomi Mi 10 Lite mobile phone was used as the experimental tool, and magnetic field data were collected using the AndroSensor software to build a database. Figure 5 presents the results of Kriging interpolation. The passages between the north and south of the building had high magnetic intensity values in both the vertical and horizontal directions. As stated by Li et al. [10], a large amount of steel changes the indoor environmental magnetic field. Thus, we inferred that the connecting passage should be the location of the instrument and computer rooms. The environment contained many heavy metal utensils; thus, its magnetic force value was larger than those of the other areas. The training results were used for subsequent indoor magnetic field positioning experiments.

**Figure 6:**
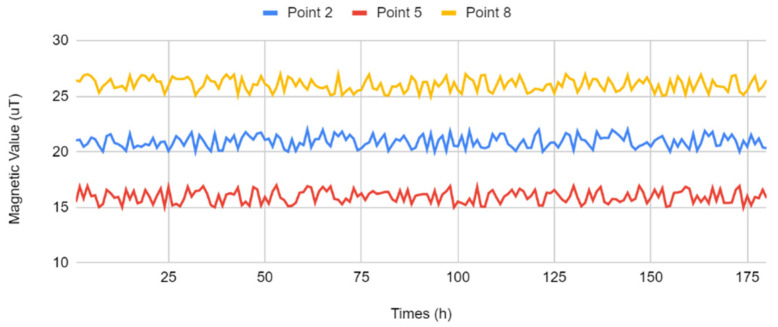
Variation in the curve of magnetic intensity values at different sampling points.

Finally, the stability of the indoor magnetic fingerprint map was analyzed. Three test points were selected as the sampling points, and the magnetic intensity value of the location was determined at different time intervals; the hourly variations in the values were analyzed. Figure 6 presents the variation in the curves of the magnetic intensities at the different sampling points. The mean magnetic intensity values at the three sampling points were 20.927, 15.970, and 26.053 μT, respectively. The standard deviations were 0.559, 0.579 and 0.580, respectively. The magnetic intensity value was relatively stable over a medium and long term, and the indoor magnetic field exhibited a fixed value; these findings were similar to those reported for the environmental magnetic fields in previous studies.

### 4.2. Analysis of Constraint Correction

**Figure 7:**
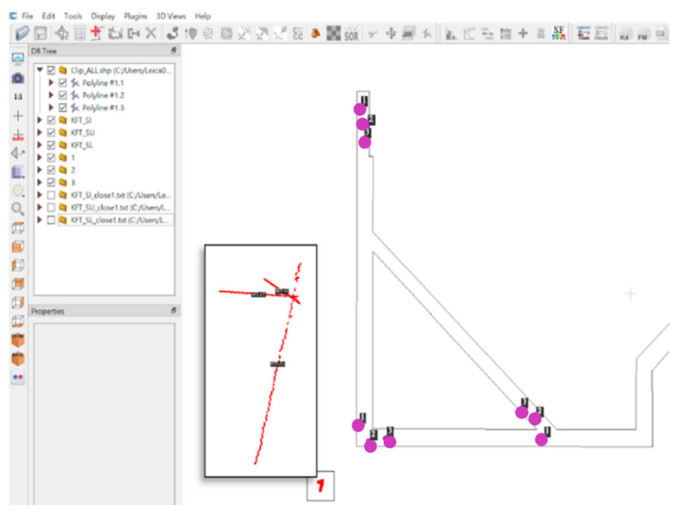
Schematic of key frames and matching points.

We minimized the positioning error to ensure that the positioning point traveled on the correct path and processed positioning data in real time. This study used the concepts of scene constraints and magnetic matching coordinates as the starting point of the ORB-SLAM line segment. The initial point was used as a scale to solve the problem of the trajectory offset caused by the rotation to complete indoor positioning coordinate correction. When the matching point of the magnetic field was used as the starting point, at least three consecutive points were required, and the three points were based on the matching coordinates obtained using the three consecutive steps. We performed a 4PCS coordinate conversion by using the positioning coordinates of the front part of the ORB-SLAM system to determine the conversion parameters. Then, the ORB-SLAM key frame coordinates were converted to real coordinates. Figure 7 presents the key frame coordinates and matching points. In Figure 7, the red points represent the SLAM key frame positions, and the purple points indicate the magnetic field matching points at the beginning of the line segments.

**Figure 8:**
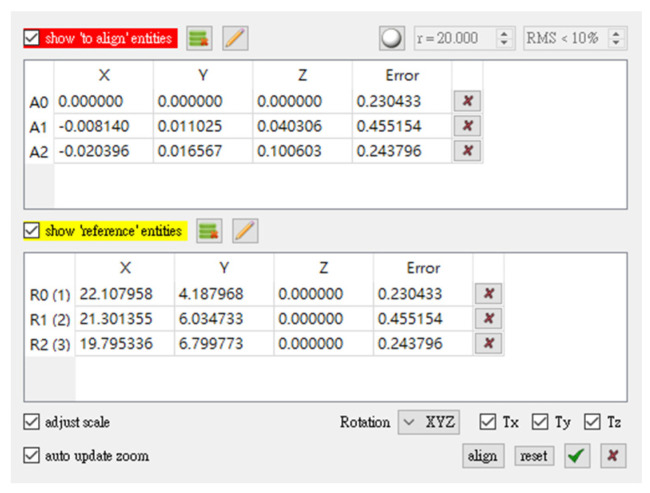
Schematic of 4PCS coordinate transformation.

**Figure 9:**
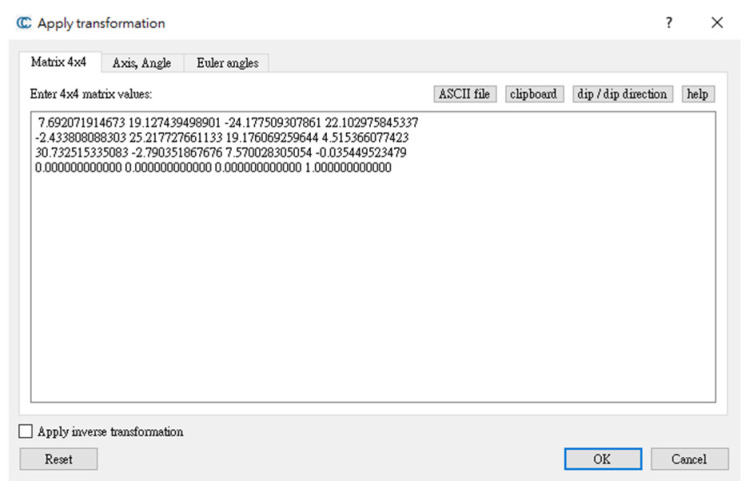
Schematic of conversion parameters.

In the ORB-SLAM coordinate system, the first image was used as the starting point (0, 0, 0). The camera center coordinates were considered as the position; these points differed from the coordinates obtained from the sixth-floor plane in this experiment by one rotation and translation. We used the coordinate transformation method to determine the transformation parameters of the two coordinate systems. Figure 8 presents a schematic of the selection of the coordinate conversion points. The magnetic field matching point was used as the reference point, and the ORB-SLAM coordinate was used as the point to be converted. As presented in Figure 9, the original ORB-SLAM coordinate trajectory was converted to obtain the movement trajectory after the coordinate constraints were corrected in the magnetic fingerprint map.

Compared with loose coupling, this constraint method had the following two advantages. Coupling weighting uses the RMSE of two or more positioning techniques as the weight. However, when the positioning accuracy of any technique was considerably low, the overall accuracy of the ORB-SLAM system was high. However, a large deviation was observed in the latter part of the trajectory, resulting in a situation where both the weight and the positioning error were high. In addition, the coupling of the overall calculation process and the check point cannot be performed immediately; the check point can only be determined on the basis of the positioning results. Different from the coordinate constraint correction method, the conversion parameters could be obtained in real time through the initial location of positioning technology, and a starting reference for the subsequent key frames could be obtained.

**Figure 10:**
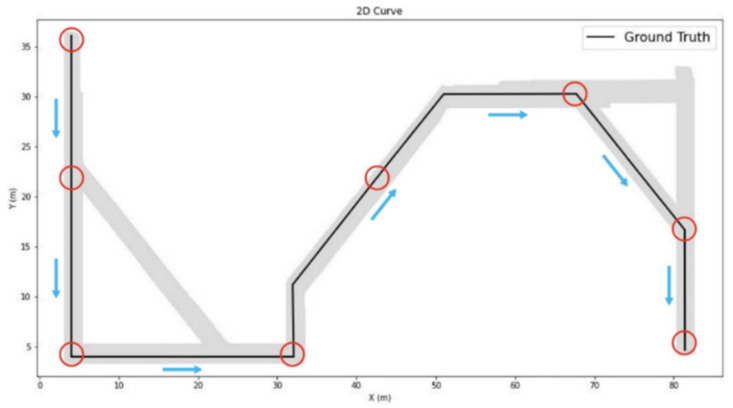
Experimental route.

The scope of the experiment included the north and south buildings with a total route length of 140 m, and eight check points were planned along the route to determine the positioning accuracy. The route included the 45° and 90° rotations. Figure 10 illustrates the distribution of the eight check points and the direction of the route used in this study.

**Figure 11:**
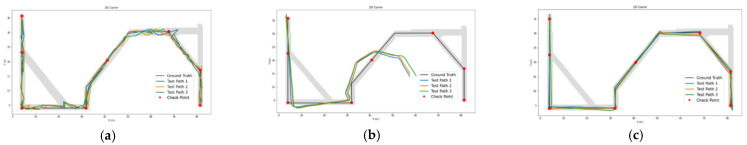
Positioning trajectory before and after coordinate constraint corrections.

Figure 11a depicts the results of magnetic field positioning. Twists and turns were observed in the course of the trajectory. Figure 11b presents the positioning trajectory of the ORB-SLAM system. The overall trajectory was not attached to the end point. In most cases, the trajectory deviated from the real path and was out of the walkable range. Figure 11c illustrates the positioning trajectory of the coordinate constraint correction method. The corrected trajectory was within the walking range. This method effectively controlled accumulation errors and, thus, can be used for indoor positioning.

**Figure 12:**
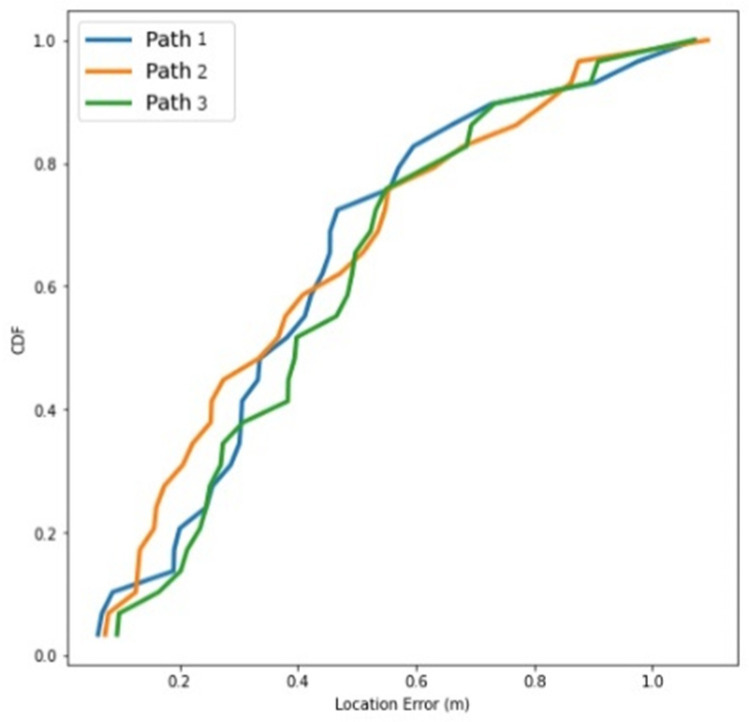
Distribution of cumulative errors in different routes.

Table 5 presents the positioning errors determined after coordinate constraint corrections. The mean error of the test data of the three sets was 0.613 m. The maximum error was approximately 1 m, and the minimum error was less than 0.1 m. The three sets of the test data had stable results (Figure 12), and the positioning error exhibited an accuracy of less than 0.6 m for 75% of the time. After long-distance movement, the algorithm used in this study exhibited good control of accumulated errors. The experimental findings indicated that the positioning accuracy could be adjusted according to different routes and user states. The level could be maintained within 1 m.

After coordinate constraint corrections, the improvements in the accuracy levels of the magnetic field and the ORB-SLAM system were 85.58% and 97.16%, respectively (Table 6). These findings indicated that the coordinate constraint correction method could effectively improve indoor positioning accuracy.

**Figure 13:**
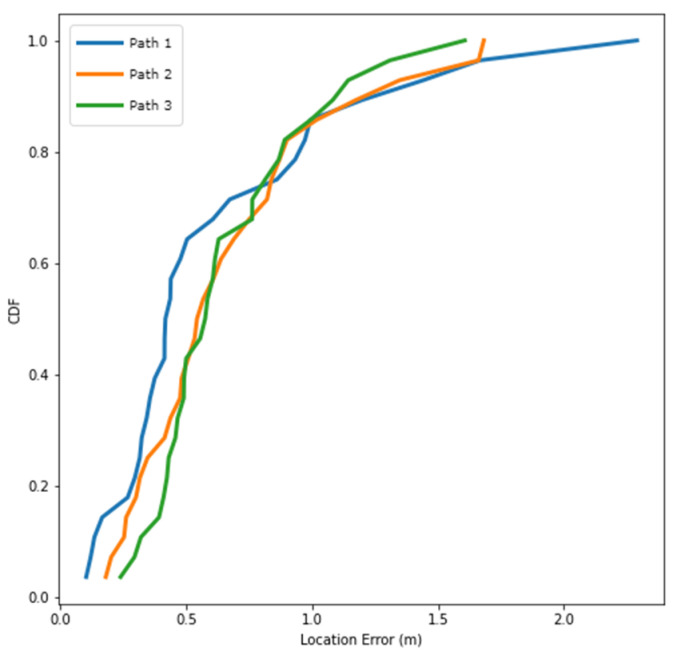
Distribution of cumulative errors in different routes (Galaxy Note 9).

To confirm the feasibility of the positioning system, we evaluated the same user at different times and states using different brands of mobile devices. The Samsung Galaxy Note 9 was selected as the second experimental device. The positioning errors of the magnetic field and the ORB-SLAM system were 1.762 m and 12.065 m, respectively (Table 7). The positioning error of the coordinate constraint corrections reached 0.697 m. The accuracy was approximately 60% higher than that of magnetic field positioning and approximately 95% higher than that of the ORB-SLAM system. Figure 13 reveals that in 80% of cases, the errors could be maintained below 1 m, and only some cases had errors of more than 1.5 m.

The positioning results and walking trajectories of a single positioning technology considerably differed from different mobile devices, and no correlation was observed between the positioning results and walking trajectories. However, the results obtained without correction could not meet the required indoor positioning accuracy. The positioning results obtained after coordinate constraint corrections were relatively stable. The positioning could still be determined within the same indoor range, and the positioning accuracy was between 0.5 and 0.7 m even when different mobile devices were used. Therefore, desirable positioning results were obtained for the same user by using different mobile devices at different times and states.

## 5. Discussion and Conclusions

In this study, magnetic fingerprint map positioning was used to determine the initial location of a user. Approximate coordinates were determined through the ORB-SLAM system. Subsequently, the coordinate constraint correction method was used to correct the trajectory error, thus reducing the distance error caused by error accumulation and the determination of indoor positions. We summarized the findings of this study and proposed suggestions for future research:

1. Magnetic field positioning has the advantage of a low cost, and the fingerprint map has a sufficient stability. With the use of different algorithms, the success rate and stability of matching can be increased. In this study, square grid path planning was used to collect the magnetic field data, and the Kriging interpolation and WKNN deterministic matching methods were used to obtain the magnetic field positioning results of the initial coordinate of the user in a short time. From the magnetic fingerprint map, the user′s coordinates could be obtained, and the positioning accuracy could still maintain the required standard.
2. ORB-SLAM shooting could improve positioning stability and prevent a large trajectory offset in the later stage by planning paths in advance to ensure the closeness of trajectories to each other when a closed route is being shot. Furthermore, the shooting images were mainly obtained from a short distance. When the target was far away, there was only rotation, and there was no parallax between images, resulting in sparse keyframe generation and decreased accuracy. Finally, pure rotation movement was avoided, and half of the feature points in each image were obtained from the previous image.
3. This study combined the advantages of VSLAM and a magnetic fingerprint map to improve the accuracy of indoor positioning. Magnetic matching coordinates were used as the initial coordinates of the ORB-SLAM route as well as a scale basis to overcome the weakness of a single positioning technology. The findings indicated that the accuracy of positioning can be improved from a range of 1.5 to 2 m to a range of 0.5 to 0.7 m through the proposed method, and the same positioning accuracy could be achieved by using this method with mobile devices of different brands. Therefore, this method demonstrated strong positioning effectiveness and reliability and can be used as an alternative method for indoor positioning.
4. Despite the fact that this study confirmed that the proposed method can effectively improve indoor positioning accuracy, the positioning system only applies to the relative coordinate system. The model only solved the collected data and conversion parameters in the computer through postprocessing. The research results still need to be developed to be able to be executed in the absolute coordinate system on mobile devices to achieve real-time indoor positioning. There are opportunities to create and apply subsequent applications based on the constraint method proposed in this study in the future.

## Figures and Tables

**Table 1:** Comparison of coupling methods.

Method	Way	Relation	Sensor	Scale
Tightly coupling	simultaneous	strong	centralized	small
Loosely coupling	nonsimultaneous	weak	decentralized	large

**Table 2:** Technical comparison of coupling systems.

Positioning System	Method	Accuracy (m)	Scale
MSF [16]	EKF	0.350	5 m^3^
VMag [17]	Environment-aware Particle Filter	1.095	2000 m^2^
ORB-SLAM+IMU [18]	LKF	0.140	18 m^2^
ORB-SLAM3 [19]	Optimized image Location and Orientation	0.043	250 m

**Table 3:** Specifications of Xiaomi Mi 10 Lite.

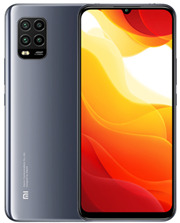	Supplier	Xiaomi Corporation
	Headquarters	Beijing, China
	Size	163.91×74.77×7.98 (mm)
	Display	6.57” AMOLED TrueColor
	CPU	Qualcomm Snapdragon 765 G 2.4 GHz Octa-core processor
	OS	Android 10
	Sensors	Accelerometer, Gyroscope, Magnetometer
	Camera	Rear: 48 million pixels, 1/2” ultra-large sensor, f/1.79 ultra-large aperture
		Front: 16 million pixels, 1080P (30 fps)

**Table 4:** Calibration parameters (Mi 10 Lite).

Element of Interior Orientation/Standard Deviation		Test 1	Test 2	Test 3
Camera Interior Orientation Elements	f (pixel)	653.135/0.346	655.121/0.371	653.189/0.351
	$x_{p}$ (pixel)	314.571/0.646	316.008/0.610	315.486/0.597
	$y_{p}$ (pixel)	240.144/0.592	240.713/0.549	240.521/0.514
Radial distortion parameters	$K_{1}$	0.114/0.0027	0.161/0.0024	0.085/0.0019
	$K_{2}$	−0.468/0.0108	−0.715/0.0098	−0.804/0.00103
	$K_{3}$	0.372/0.0002	0.422/0.0001	0.561/0.0001
Decentering distortion parameters	$P_{1}$	0/0	0/0	0/0
	$P_{2}$	0/0	0/0	0/0

**Table 5:** Positioning error after coordinate constraint corrections (m).

	Min	Max	Mean	RMSE
1	0.062	1.072	0.603	0.693
2	0.074	1.093	0.613	0.683
3	0.094	1.069	0.622	0.678
Mean	0.076	1.078	0.613	0.685

**Table 6:** Comparison of the positioning results of different methods.

	Magnetic Field		ORB-SLAM		Constraint
	Mean Error (m)	Improvement %	Mean Error (m)	Improvement %	Mean Error (m)
1	4.264	85.86	20.790	97.10	0.603
2	4.411	86.10	21.774	97.18	0.613
3	4.067	84.71	22.071	97.18	0.622
Mean	4.247	85.58	21.545	97.16	0.613

**Table 7:** Comparison of positioning results using different methods (Galaxy Note 9).

	Magnetic Field		ORB-SLAM		Constraint
	Mean Error (m)	Improvement %	Mean Error (m)	Improvement %	Mean Error (m)
1	1.746	63.40	12.217	94.77	0.639
2	1.672	55.76	12.392	94.03	0.740
3	1.868	61.93	11.585	93.86	0.711
Mean	1.762	60.47	12.065	94.23	0.697

## Data Availability

Not applicable.
